# Management of Patients with Type V Hyperlipoproteinemia: An Uncommon Phenotype of Dyslipidemia with Chylomicronemia and Severe Hypertriglyceridemia

**DOI:** 10.3390/jpm13010068

**Published:** 2022-12-28

**Authors:** Ya-Hui Chang, Dai-Yi Lin, Chia-Ling Tsai, Chih-Hung Liang, Yu-Ting Yu, Yi-Lin Hsieh, Jen-Yu Chuang, Yi-Han Chen, Hung-I Yeh, Chao-Feng Lin

**Affiliations:** 1 Department of Medicine, MacKay Medical College, New Taipei City 252005, Taiwan; 2 Department of Pharmacy, MacKay Memorial Hospital, Taipei 104217, Taiwan; 3 Department of Cardiology, MacKay Memorial Hospital, Taipei 104217, Taiwan; 4 Department of Medical Education, MacKay Memorial Hospital, Taipei 104217, Taiwan; 5 School of Public Health, College of Public Health, Taipei Medical University, Taipei 110301, Taiwan

**Keywords:** type IV hyperlipoproteinemia, type V hyperlipoproteinemia, hypertriglyceridemia

## Abstract

Hypertriglyceridemia (HTG) remains a risk-enhancing factor of atherosclerotic cardiovascular disease. We aimed to report real-world data on the management of patients with type V hyperlipoproteinemia (HLP5), an uncommon phenotype of dyslipidemia characterized by fasting chylomicronemia and severe HTG. Between July 2018 and May 2021, 90 patients with HTG, including 83 patients with type IV hyperlipoproteinemia (HLP4) and 7 patients with HLP5, were identified by plasma apolipoprotein B (apoB) and lipoprotein electrophoresis. Patients with HLP5 were younger, had higher total cholesterol (TC) (264.9 ± 26.7 mg/dL vs. 183.9 ± 26.1 mg/dL; *p* < 0.01) and higher triglyceride (TG) (1296.7 ± 380.5 mg/dL vs. 247.6 ± 96.1 mg/dL; *p* < 0.01), and had lower high-density lipoprotein cholesterol (HDL-C) (30.6 ± 4.8 mg/dL vs. 40.5 ± 8.7 mg/dL; *p* < 0.01) and lower low-density lipoprotein cholesterol (LDL-C) (62.9 ± 16.4 vs. 103.0 ± 21.1 mg/dL; *p* < 0.01) compared with patients with HLP4. Despite an aggressive use of statin and fenofibrate with greater reductions in TG (-65.9 ± 13.7% vs. -27.9 ± 30.5%; *p* < 0.01) following 6 months of treatment, patients with HLP5 had persistent HTG (440.1 ± 239.0 mg/dL vs. 173.9 ± 94.8 mg/dL; *p* < 0.01) and an increase in LDL-C (28.3 ± 57.2% vs. -19.5 ± 32.0%; *p* < 0.01) compared with patients with HLP4. Our findings highlight that the lack of novel TG-lowering medications and management guidelines remains an unmet medical need in patients with HLP5. Closely monitoring lipid profiles, full assessment of individual’s risk of cardiovascular disease, and emphasis on medication adherence are of clinical importance.

## 1. Introduction

By using lipoprotein electrophoresis (LPEP) and ultracentrifugation, Fredrickson, Levy, and Lees (FLL) separated clinical dyslipidemia into five phenotypes (types I–V) of hyperlipoproteinemia (HLP) [1], each phenotype represented a characteristic abnormality of specific lipoproteins. Although the World Health Organization (WHO) adopted this classification [2], the current lipid guidelines [3,4] have been simplified to focus on the elevation of low-density lipoprotein cholesterol (LDL-C) in clinical practice. Recently, Sniderman et al. validated a diagnostic apolipoprotein B (apoB) algorithm derived from patients with genetically verified dyslipidemia to determine the FLL phenotypes of HLP by evaluating the conventional lipid profiles together with the plasma apoB levels [5]. The apoB algorithm can provide valuable information to indicate which lipoprotein abnormalities are involved: chylomicrons (types I and V), very low-density lipoproteins (VLDLs) (types IIb, IV, and V), low-density lipoproteins (LDLs) (types IIa, IIb), or remnant lipoproteins (type III) [6,7,8]. According to the apoB algorithm, type IIa (HLP2a) and IIb (HLP2b) are identified by apoB ≥ 120 mg/dL together with variable plasma TG levels, while type I (HLP1), III (HLP3), IV (HLP4), and V (HLP5) are identified by apoB < 120 mg/dL with moderately to severe hypertriglyceridemia (HTG) [5]. The use of the apoB algorithm may assist in assessing the possible pathogenic mechanisms of each FLL phenotype and the residual cardiovascular risk of patients who have elevated TG-rich atherogenic lipoproteins but a normal plasma LDL-C level [5].

HTG is associated with a decrease in high-density lipoprotein cholesterol (HDL-C) and an increase in atherogenic lipoproteins. Previous epidemiological and Mendelian randomization studies have demonstrated that the risk of atherosclerotic cardiovascular disease (ASCVD) remains high in patients with HTG despite statin therapy [9,10,11,12]. In addition, moderate to severely elevated plasma TG is associated with an increased risk of pancreatitis [3]. Based on the available evidence, the current American lipid guidelines have recommended the identification of HTG as a “risk-enhancing factor” of ASCVD [3], and the use of TG-lowering medications is also recommended in patients with moderate to severe HTG [3]. Of the HTG-associated phenotypes of HLPs, HLP4 and HLP5 are both referred to as polygenic lipid disorders complicated with various degrees of metabolic derangements accompanied by a marked increase in plasma VLDLs [1,2,5]. Specifically, the identification of HLP5 is separated from HLP4 by the presence of chylomicrons in the fasting blood, which can be determined by LPEP [1,5,6]. HLP4 is the most common FLL phenotype with HTG, whereas the prevalence of HLP5 in the adult population is uncommon [6]. Additionally, it is difficult to differentiate HLP5 from HLP4 by conventional blood lipid tests without the apoB algorithm and LPEP, leading clinicians to easily ignore HLP5 in clinical practice [6]. Until now, evidence showing the management of patients with HLP5 remains scarce. The present study aimed to report real-world data on the management of patients with HLP5 and persistent HTG.

## 2. Materials and Methods

### 2.1. Study Design

The present study was designed as a retrospective analysis of prospectively-enrolled patients. It was conducted in the Lipid Clinic of our institution, established since July 2018. The aim of the Lipid Clinic is to provide uniform standards of diagnosis and treatment for patients with lipid disorders based on the current lipid guidelines, regulation of national health insurance, and full physician–patient discussion [13]. All of the protocols of the present study were approved by the Institutional Review Board of our institution (approval no. 18MMHIS083e), and written informed consent was obtained from all participants. Venous blood samples of patients who visited the Lipid Clinic were collected after 12 h of fasting for biochemistry examination, blood lipid tests (i.e., total cholesterol (TC), TG, LDL-C, HDL-C, and apoB), LPEP, serum albumin levels, and thyroid function tests.

### 2.2. Lipoprotein Electrophoresis (LPEP)

The blood samples were collected into serum-separating tubes. After a brief centrifugation (2000× *g*, 30 min) to remove the residual debris of blood cells, the supernatant was prepared for LPEP by isoelectric focusing methods using a commercial kit (Quick gel, Helena Laboratories, Saitama, Japan). Briefly, 30 μL of each blood sample was loaded onto the agarose gel for migration at 250V and 20 °C. Lipoproteins in the blood samples were separated into four major bands on agarose gels based on their electric charges: chylomicron, VLDL (pre-β lipoprotein), LDL (β-lipoprotein), and HDL (α-lipoprotein) [14]. Each band was assessed and analyzed by densitometric scanning by using Edbank III analysis software (Helena Laboratories, Saitama, Japan) to determine the proportion of each lipoprotein fraction. The presence of chylomicrons in the fasting blood determined by LPEP was an important characteristic feature of HLP5, whereas chylomicrons in the fasting blood was absent in HLP4 [1,5,6].

### 2.3. Identification of Each FLL Phenotype by apoB Algorithm and LPEP

The patients’ FLL phenotypes of HLP were determined by fasting lipid parameters, apoB levels [1,5,6], and LPEP (Supplementary Table S1). Briefly, the diagnostic criteria of each FLL phenotype were as follows: (1) HLP1: apoB < 75 mg/dL, TG ≥ 150 mg/dL, and TG/apoB ratio ≥ 8.8; (2) HLP2a: apoB ≥ 120 mg/dL, TG < 150 mg/dL, and an absence of chylomicrons on LPEP; (3) HLP2b: apoB ≥ 120 mg/dL, TG ≥ 150 mg/dL, and an absence of chylomicrons on LPEP; (4) HLP3: apoB < 120 mg/dL, TG/apoB ratio < 8.8, TC/apoB ratio ≥ 2.4, and the presence of broad-β band on LPEP; (5) HLP4: apoB < 120 mg/dL, TG ≥ 150 mg/dL and TG/apoB ratio < 8.8, TC/apoB ratio < 2.4, and the absence of chylomicrons on LPEP; (6) HLP5: apoB < 120 mg/dL and ≥75 mg/dL, TG ≥ 150 mg/dL, TG/apoB ratio ≥ 8.8, and the presence of chylomicrons on LPEP [1,5,6].

Based on the abovementioned criteria, no patients with HLP1 or HLP3 were identified during the study period. Patients with HLP4 or HLP5 who had HTG (defines as fasting plasma TG levels ≥150 mg/dL) [15] were identified and prospectively enrolled in the present study. Patients who were less than 20 years old; unable to receive regular blood examination; disagreed with providing personal medical information; had histories of thyroid dysfunction, nephrotic syndrome, and malignancy; had received regular dialysis; had elevated liver enzymes; and had received steroid, immunotherapy, antiviral agents, and hormone replacement therapy were excluded. Patients who had a serum albumin level < 2.5 g/dL or abnormal thyroid function tests at baseline were also excluded. Finally, patients were divided into two groups: patients with HLP4 and patients with HLP5.

### 2.4. Data Collection

After enrollment, specially-trained medical staff reviewed the patients’ medical records and collected all baseline data whenever feasible, including age, sex, weight, height, smoking habits, histories of comorbidities, laboratory data, and concurrent prescribed medications. Body mass index was defined as weight in kilograms was divided by the square of height in meters. Patients with coronary artery disease (CAD) were defined as those who had >50% diameter stenosis of major epicardial coronary arteries confirmed by coronary computed tomography (CT) angiography or coronary angiography. The identification of ischemic stroke (IS) was based on the neurologist’s records with confirmation by brain CT or magnetic resonance imaging. Diagnosis of peripheral arterial disease (PAD) was confirmed by an ankle-brachial index <0.9 or >1.4 and/or >50% diameter stenosis of peripheral arteries observed in CT angiography. Diagnosis of diabetes mellitus (DM) and hypertension (HTN) were based on medical records and prescribed medications. The history of pancreatitis was identified by patients’ admission records together with the detection of raised circulating pancreatic enzyme and pancreatic CT scans (Table S2).

Prescribed medications, including antiplatelet, beta-blocker, and angiotensin converting enzyme inhibitor/angiotensin receptor blocker (ACEI/ARB), were recorded. The lipid-lowering therapy (LLT) of each patient, including statin, ezetimibe, proprotein convertase subtilisin/kexin type 9 (PCSK9) inhibitor, fenofibrate, niacin, and omega-3 fatty acids, was also recorded (Table S2). Atorvastatin 40–80 mg/day and rosuvastatin 20 mg/day were referred to as high-intensity statins (HISs) [3].

Each participant in the present study received initiation or adjustment of LLT according to the actual clinical situation, physician–patient discussions, and current regulation of the national health insurance. At each visit, the patients’ prescribed LLT and fasting lipid profiles, including TC, TG, LDL-C, HDL-C, non-HDL-C, and apoB, were recorded and analyzed. The plasma non-HDL-C level was calculated as TC minus HLD-C. In addition, persistent HTG was defined as fasting TG ≥ 150 mg/dL following a minimum of 4 to 12 weeks of maximally tolerated statin therapy when indicated according to the “2021 ACC Expert Consensus Decision Pathway on the Management of ASCVD Risk Reduction in Patients with Persistent Hypertriglyceridemia” [15].

### 2.5. Study Outcomes

The study outcomes of the present study were patients’ fasting plasma TC, TG, LDL-C, HDL-C, non-HDL-C, and apoB levels; the percentages of change in each lipid parameter; and the prescription rates of LLT at the 6-month follow-up.

### 2.6. Statistical Methods

Categorical and continuous variables were each presented with numbers (percentages) and means (±standard deviations). Comparisons were performed using the chi-square or Fisher’s exact test for categorical variables, as appropriate, and the unpaired Student t test or Wilcoxon rank sum test for continuous variables. The baseline characteristics of patients with HLP5 were compared with patients with HLP4. In addition, the patients’ fasting plasma TC, TG, LDL-C, HDL-C, non-HDL-C, and apoB; the percentages of change in each lipid parameter; and the prescription rates of LLT at the 6-month follow-up were compared between groups. Significance was set at *p* < 0.05 (two-tailed). SAS statistical software (version 9.2 for Windows; SAS Institute, Cary, NC, USA) was used for all analyses.

## 3. Results

### 3.1. Baseline Demographic Characteristics of Patients

Between July 2018 and May 2021, 90 patients who had fasting HTG, including 83 patients with HLP4 and 7 patients with HLP5, were identified. Patients with HLP5 were younger (41.6 ± 10.5 years vs. 57.4 ± 10.0 years; *p* < 0.01), had a higher body mass index (31.4 ± 2.2 kg/m^2^ vs. 27.2 ± 3.7 kg/m^2^; *p* < 0.01), and had less hypertension (14.3% vs. 67.5%; *p* < 0.01) and more pancreatitis (14.3% vs. 0%; *p* = 0.08) (Table 1).

Patients with HLP5 had higher glomerular filtration rates (100.5 ± 14.8 mL/min vs. 77.4 ± 27.9 mL/min; *p* = 0.01), higher TC (264.9 ± 26.7 mg/dL vs. 183.9 ± 26.1 mg/dL; *p* < 0.01), higher TG (1296.7 ± 380.5 mg/dL vs. 247.6 ± 96.1 mg/dL; *p* < 0.01), and higher non-HDL-C (234.3 ± 26.6 mg/dL vs. 143.4 ± 24.7 mg/dL; *p* < 0.01) levels, while they had lower LDL-C (62.9 ± 16.4 mg/dL vs. 103.0 ± 21.1 mg/dL; *p* < 0.01), lower HDL-C (30.6 ± 4.8 mg/dL vs. 40.5 ± 8.7 mg/dL; *p* < 0.01), and similar apoB (94.5 ± 10.2 mg/dL vs. 101.6 ± 13.5 mg/dL; *p* = 0.09) levels compared with patients with HLP4. The baseline prescription rates of LLT were similar between groups, except patients with HLP5 received statin therapy less frequently (14.3% vs. 61.4%; *p* = 0.04) compared with the patients with HLP4 (Table 1).

### 3.2. The Use of LLT and Lipid Parameters at the 6-Month Follow-Up

**Figure 1:**
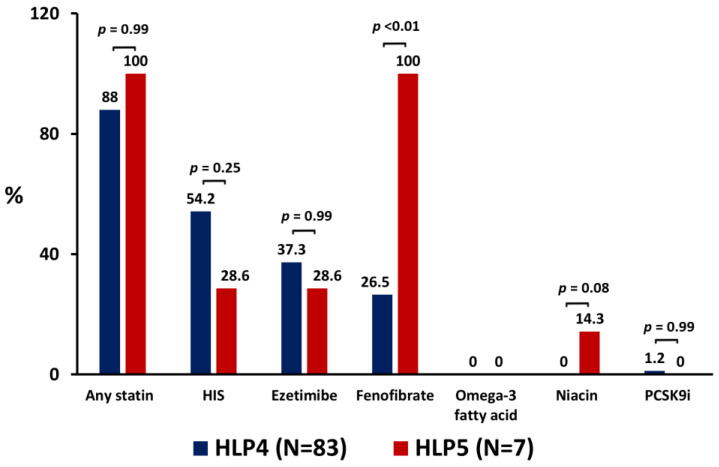
Prescription rates of LLT in patients with HLP4 and patients with HLP5 at the 6-month follow-up. HIS = high-intensity statin; HLP4 = type IV hyperlipoproteinemia; HLP5 = type V hyperlipoproteinemia; LLT = lipid-lowering therapy; *N* = number; PCSK9i = proprotein convertase subtilisin/kexin type 9 inhibitor (*p* value was calculated as HLP5 vs. HLP4).

The prescription rates of any statin, HIS, ezetimibe, and fenofibrate at the 6-month follow-up were increased among patients with HLP5 and patients with HLP4 in the present study. Notably, all patients with HLP5 received statin in combination with fenofibrate therapy. Compared with patients with HLP4, patients with HLP5 had more frequently received fenofibrate (100% vs. 26.5%; *p* < 0.01) compared with patients with HLP4. In addition, there was also a trend of increased use in niacin among patients with HLP5 than patients with HL4, but this trend did not reach a statistical significance (Figure 1). The use of omega-3 fatty acids was very limited in patients with HLP5 and patients with HLP4. There were no drug-associated adverse events, including elevated liver enzymes, creatine kinase, myalgia, myopathy, and rhabdomyolysis, reported by patients in the present study.

**Figure 2:**
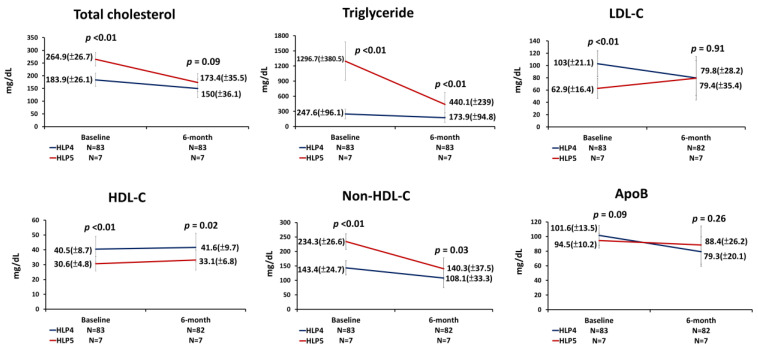
The changes of lipid parameters from baseline to the 6-month follow-up in patients with HLP4 and patients with HLP5. ApoB = apolipoprotein B; HDL-C = high-density lipoprotein cholesterol; HLP4 = type IV hyperlipoproteinemia; HLP5 = type V hyperlipoproteinemia; LDL-C = low density lipoprotein cholesterol; N = number; Non-HDL-C = non-high-density lipoprotein cholesterol (p value was calculated as HLP5 vs. HLP4).

There were no patients lost follow-up during the 6-month observation. Only one patient with HLP4 had no data of plasma LDL-C, HDL-C, and apoB levels at the 6-month follow-up. Following 6 months of treatment, patients with HLP5 had similar TC (173.4 ± 35.5 mg/dL vs. 150.0 ± 36.1 mg/dL; *p* = 0.09), LDL-C (79.4 ± 35.4 mg/dL vs. 79.8 ± 28.2 mg/dL; *p* = 0.91), and apoB (88.4 ± 26.2 mg/dL vs. 79.3 ± 20.1 mg/dL; *p* = 0.26) levels compared with patients with HLP4 (Figure 2). However, patients with HLP5 still had persistently higher TG (440.1 ± 239.0 mg/dL vs. 173.9 ± 94.8 mg/dL; *p* < 0.01), lower HDL-C (33.1 ± 6.8 mg/dL vs. 41.6 ± 9.7 mg/dL; *p* = 0.02), and higher non-HDL-C (140.3 ± 37.5 mg/dL vs. 108.1 ± 33.3 mg/dL; *p* = 0.03) levels compared with patients with HLP4 (Figure 2).

### 3.3. Percentages of Change in Lipid Profiles at the 6-Month Follow-Up

**Figure 3:**
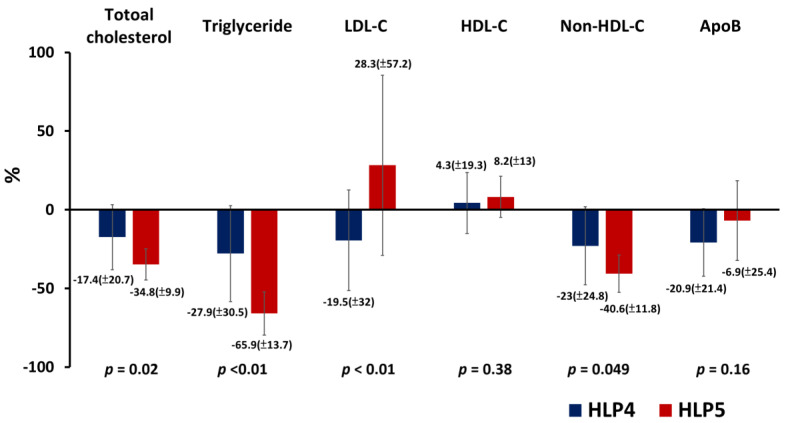
Percentage changes in each lipid parameter following the 6-month treatment in patients with HLP4 and patients with HLP5. ApoB = apolipoprotein B; HDL-C = high-density lipoprotein cholesterol; HLP4 = type IV hyperlipoproteinemia; HLP5 = type V hyperlipoproteinemia; LDL-C = low density lipoprotein cholesterol; *N* = number; Non-HDL-C = non-high-density lipoprotein cholesterol (*p* value was calculated as HLP5 vs. HLP4).

After 6 months of treatment, patients with HLP5 had a significantly more reduction in plasma TC (−34.8 ± 9.9 vs. −17.4 ± 20.7%; *p* = 0.02), TG (−65.9 ± 13.7 vs. −27.9 ± 30.5%; *p* < 0.01), and non-HDL-C (−40.6 ± 11.8 vs. −23.0 ± 24.8%; *p* = 0.049) compared with patients with HLP4 (Figure 3). However, patients with HLP5 had a significant increase in plasma LDL-C (28.3 ± 57.2 vs. −19.5 ± 32.0%; *p* < 0.01) and an insignificant reduction in apoB (−6.9 ± 25.4 vs. −20.9 ± 21.4%; *p* = 0.16) compared with patients with HLP4 (Figure 3).

## 4. Discussion

The present study showed that the demographic characteristics of patients with HLP5 were quite different from those of patients with HLP4. Despite an aggressive use of statin and TG-lowering medications followed by a marked reduction in plasma TC, TG, and non-HDL-C, patients with HLP5 still stayed in persistent HTG and had an incremental change in plasma LDL-C without a significant alteration in plasma apoB compared with patients with HLP4. To the best of our knowledge, the present study is the first evidence showing a real-word data on the management of patients with HLP5.

HLP5 is scarcely investigated, with a prevalence of 0.13-0.15% in the adult population, while HLP4 is the most common phenotype of HLP with a prevalence of 20.5-24.1% [6]. It is impossible to separate HLP5 from HLP4 based on conventional blood lipid tests, which usually results in a misdiagnosis of HLP5. An important distinguishing feature to identify HLP5 is the presence of chylomicrons together with an elevation in VLDLs in the fasting blood samples, whereas chylomicrons are absent in HLP4 [1,5]. The findings in the present study implicate that the use of LPEP in combination with the apoB algorithm may increase the diagnostic accuracy and assist in the differentiation between HLP4 and HLP5. Additionally, blood samples collected following at least 10 h of fasting could increase the accuracy of identification in plasma chylomicrons [1,5,6]. The pathogenic mechanisms of HLP5 remains not fully understood. It has been reported that patients with HLP5 may carry heterozygous mutations in *LPL*, *GHIHBP1*, *APOC2*, *APOA5*, or *LMF1* genes, or burdens of single nucleotide polymorphisms in multiple TG-associated genes (i.e., *DOCK7*, *ANGPTL3*, *GALNT2*, *KLHL8*, *AFF1*, *MAP3K1*, *ANKRD55*, *MLXIPL*, *NAT2*, *TRIB1*, *JMJD1C*, *FADS1*, *FADS2*, *FADS3*, *APOA1*, *APOC3*, *APOA4*, *CAPN3*, *FRMD5*, *CETP*, *SUGP1*, *PLTP*, *GCKR*, *CYP26A1*, or *CILP2*) accompanied by secondary metabolic or environmental factors (i.e., obesity, alcohol drinking, or diabetes) compared with patients with HLP4, resulting in a further decreased activity in lipoprotein lipase (LPL), reduced clearance of chylomicrons and VLDLs, and further elevation of plasma TG levels [16,17].

As plasma TG levels rise with a decrease in LPL activity, the circulating VLDL particles increase, accompanied by inefficient lipolysis and impaired clearance [18]. These VLDLs and their remnant particles enriched in apolipoprotein E and apolipoprotein C-III (apoC-III) may retain in the arterial wall, contributing to the initiation and progression of atherosclerosis, vascular inflammation, and prothrombotic effects [19]. HTG also results in the formation of small, dense LDLs that have a high atherogenicity because of their longer residence time in circulation and bioactive inflammatory lysolipids [20]. Moderate to severe HTG also leads to an increased risk of pancreatitis [15]. The excess of TG hydrolyzed by pancreatic lipase released in the vascular bed of the pancreas leads to high concentrations of free fatty acids, which overwhelm the binding capacity of albumin and self-aggregate to micellar structures with detergent properties, thereby exhibiting a toxic injury to pancreatic acinar cells. Additionally, the elevated levels of TG-rich lipoproteins increase the viscosity of blood and thus impair the blood flow in the pancreas, which enhances the toxicity of free fatty acids and triggers ischemic injury within the pancreas [21].

In addition to difficult recognition and identification of HLP5 in clinical scenarios, the findings of the present study also highlight that the clinical treatment guidelines for patients with HLP5 still remain lacking. Weight loss is considered the most effective nonpharmacological intervention to lower plasma TG levels [15]. Although the response may be variable, a 5-10% reduction in body weight is associated with a 20% decrease in plasma TG [22]. Dietary recommendations should be individualized based on fasting plasma TG levels. In most circumstances, referral to a registered dietician nutritionist is necessary. Generally, the elimination of added sugar, restriction of fat to less than 15% of daily calories, and complete abstinence of alcohol are strongly advised for patients who have a plasma TG level ≥1000 mg/dL [15], as observed in patients with HLP5 in the present study. The initiation or intensification of statin therapy, together with the use of omega-3 fatty acids [15,23,24], is considered in high-risk patients with HTG who have histories of ASCVD, diabetes, or have a 10-year ASCVD risk >5%. According to the Reduction of Cardiovascular Events with Icosapent Ethyl Intervention Trial (REDUCE-IT) [24], which enrolled 8179 patients who mostly had received statin therapy, patients who were randomized to 4 g of omega-3 fatty acids (in the form of icosapent ethyl) had a 21.6% reduction in plasma TG levels from baseline and a reduced risk of composite cardiovascular events compared with patients who were randomized to the placebo. Fenofibrate is also effective to lower plasma TG; nevertheless, it did not exhibit definite clinical benefits in reducing adverse cardiovascular events. [15,25,26] In the Action to Control Cardiovascular Risk in Diabetes (ACCORD) lipid trial, fenofibrates, in addition to statin therapy, showed a 20-30% reduction in plasma TG among patients with type 2 DM, but yielded no significant additional benefit in the primary outcome of nonfatal MI, nonfatal stroke, or death from cardiovascular causes [25]. Similarly, the Fenofibrate Intervention and Event Lowering in Diabetes (FIELD) study also showed that patients with type 2 DM randomized to fenofibrate therapy had a further 29% reduction in plasma TG, without a significant reduced risk in coronary events compared with those patients randomized to the placebo over a 5-year follow-up [26].

The cost of omega-3 fatty acids was not reimbursed by our national health insurance, leading to a very limited use in the present study. Despite an aggressive use of statin and fenofibrate resulting in a significant reduction of plasma TG from baseline, patients with HLP5 in the present study still had an incremental change in plasma LDL-C and persistent HTG, which led to an increased risk of ASCVD and pancreatitis. It has been reported that the paradoxical increase in LDL-C following TG-lowering treatment is more frequently observed in patients who have lower LDL-C, higher TG, and higher non-HDL-C before treatment [27,28]. The use of fenofibrate may enhance the activities of LPL and hepatic lipase, thereby promoting the catabolism of VLDLs and lipolytic conversion of VLDLs to LDLs [28,29], which may explain the findings in the present study. In the present study, we also observed that patients with HLP5 had a greater reduction in VLDL cholesterol (VLDL-C) compared with patients with HLP4 (Figure S1), implicating an improvement in LPL and hepatic lipase activities following treatment. Nevertheless, the paradoxical increase in LDL-C following TG-lowering treatment may lead clinicians and patients to doubt not only the efficacy of treatment, but also the potential increased risk of ASCVD. In addition to an incremental change in plasma LDL-C, patients with HLP5 did not have a marked reduction in plasma apoB, despite having significant reductions in plasma TG and non-HDL-C. These findings support that TG-lowering medications in combination with the simultaneous use or intensification of statin therapy should be implemented in patients with HLP5, although they were younger and had less comorbidities deemed to have a lower risk of ASCVD compared with patients with HLP4. Moreover, a personized risk assessment, including family history of premature ASCVD, chronic inflammatory diseases, other biomarkers (i.e., lipoprotein (a) and high-sensitivity C-reactive protein), and the presence of subclinical atherosclerosis detected by carotid ultrasound scan and coronary artery calcium scanning, should be also considered to guide TG-lowering treatment and physician–patient discussion [15].

Collectively, the development of novel pharmacological therapies is needed to treat patients with HLP5. Some medications targeting apoC-III, volanesorsen, and olezarsen have shown impressive TG-lowering effects. Volanesorsen exhibited a marked reduction in plasma apo C-III and TG (−84.2% and −76.5%, respectively) in patients with familial chylomicronemia syndrome [30]. However, a significant increase in plasma LDL-C (135%) following volanesorsen treatment was observed, potentially based on the modification of lipoprotein metabolism and increased lipolytic conversion of VLDLs to LDLs, as mentioned before [28,29]. Treatment with olezarsen was shown to provide a significant reduction in plasma TG (-56% to -60%) with variable LDL-C changes (-6% to 16%) in patients with ASCVD, familial chylomicron syndrome, and severe HTG [31]. Further clinical trials are necessary to explore the roles of pharmacological inhibition in apoC-III in patients with HLP5.

The present study was subjected to some limitations. First, the patient population was relatively small because of its single-center study design and the uncommon prevalence of patients with HLP5. In addition, the apoB algorithm was not previously validated in Taiwanese cohorts; nevertheless, the use of LPEP in the present study may assist the identification of each HLP phenotype. We also acknowledge that the detection of fasting chylomicronemia might be influenced by commercial analyzers; however, the methods used in the present study have been widely described. Second, potential changes in diet and body weight of patients during the study period, which might influence the plasma lipid levels of patients, were not surveyed. Furthermore, we did not routinely conduct LPEP in patients with HLP5 to observe the changes in the plasma chylomicrons following treatment. Third, the use of omega-3 fatty acids was very limited under the current regulation of Taiwan’s national health insurance, so the effects of omega-3 fatty acids were not shown in the present study. Finally, the present analysis did not observe and measure the patients’ clinical cardiovascular outcomes. In addition, the database of this present study did not measure and evaluate the presence of preclinical atherosclerosis (e.g., carotid intima-media thickness) and proinflammatory cytokines (e.g., high-sensitivity C-reactive protein). Future prospective and multicenter studies with a longer duration of observation are needed.

## 5. Conclusions

The present study provided a valuable clinical data showing the management of patients with HLP5. Our findings highlight that the lack of novel TG-lowering medications and management guidelines remains an unmet medical need in patients with HLP5. Closely monitoring the lipid profiles, full assessment of individual’s risk of cardiovascular disease, and emphasis on medication adherence are of clinical importance.

## Figures and Tables

**Table 1:** Baseline characteristics of patients with HLP4 and patients with HLP5.

		Total *N* = 90	HLP4 *N* = 83	HLP5 *N* = 7	*p* *
Age, mean (SD)		56.2 (10.8)	57.4 (10.0)	41.6 (10.5)	<0.01
Male, *n* (%)		64 (71.1)	57 (68.7)	7 (100.0)	0.10
BMI (Kg/m^2^)		27.5 (3.8)	27.2 (3.7)	31.4 (2.2)	<0.01
Current smoker, *n* (%)		30 (33.3)	27 (32.5)	3 (42.9)	0.68
Alcohol drinking, *n* (%)		6 (6.7)	5 (6.1)	1 (14.3)	0.39
Medical history	Hypertension	57 (63.3)	56 (67.5)	1 (14.3)	<0.01
	Diabetes	51 (56.7)	49 (59.0)	2 (28.6)	0.23
	Ischemic stroke	3 (3.3)	3 (3.6)	0 (0.0)	0.99
	CAD	35 (38.9)	34 (41.0)	1 (14.3)	0.24
	PAD	5 (5.6)	5 (6.0)	0 (0.0)	0.99
	History of pancreatitis	1 (1.1)	0 (0.0)	1 (14.3)	0.08
Prescribed medications	Antiplatelets	41 (45.6)	40 (48.2)	1 (14.3)	0.12
	ACEI/ARB	60 (66.7)	56 (67.5)	4 (57.1)	0.68
	Beta-blocker	52 (57.8)	49 (59.0)	3 (42.9)	0.45
	Any statin	52 (57.8)	51 (61.4)	1 (14.3)	0.04
	HIS	16 (17.8)	16 (19.3)	0 (0)	0.35
	Ezetimibe	7 (7.8)	7 (8.4)	0 (0.0)	0.99
	PCSK9 inhibitor	0 (0)	0 (0.0)	0 (0.0)	-
	Fibrate	11 (12.2)	11 (13.3)	0 (0.0)	0.59
	Omega-3 fatty acid	0 (0)	0 (0)	0 (0)	-
	Niacin	0 (0)	0 (0.0)	0 (0.0)	-
Laboratory data	Fasting glucose (mg/dL)	125.5 (35.5)	124.9 (34.5)	133.6 (48.9)	0.54
	HbA1c (%)	6.7 (1.2)	6.7 (1.3)	6.6 (1.3)	0.75
	Cr (mg/dL)	1.1 (0.8)	1.1 (0.8)	0.9 (0.1)	0.16
	eGFR (ml/min)	79.2 (27.7)	77.4 (27.9)	100.5 (14.8)	0.01
	TC (mg/dL)	190.2 (34)	183.9 (26.1)	264.9 (26.7)	<0.01
	TG (mg/dL)	329.2 (313.2)	247.6 (96.1)	1296.7 (380.5)	<0.01
	HDL-C (mg/dL)	39.7 (8.9)	40.5 (8.7)	30.6 (4.8)	<0.01
	Non-HDL-C (mg/dL)	150.5 (34.8)	143.4 (24.7)	234.3 (26.6)	<0.01
	LDL-C (mg/dL)	99.9 (23.4)	103.0 (21.1)	62.9 (16.4)	<0.01
	ApoB (mg/dL)	101.0 (13.4)	101.6 (13.5)	94.5 (10.2)	0.09

Abbreviation: ACEI/ARB = angiotensin-converting enzyme inhibitor/angiotensin receptor blocker; ApoB = apolipoprotein B; BMI = body mass index; Cr = creatinine; eGFR = estimated glomerular filtration rate; CAD = coronary artery disease; HDL-C = high-density lipoprotein cholesterol; HIS = high-intensity statin (HIS); HLP4 = type IV hyperlipoproteinemia; HLP5 = type V hyperlipoproteinemia; LVEF = left ventricular ejection fraction; LDL-C = low-density lipoprotein cholesterol; LLT = lipid-lowering therapy; Non-HDL-C = non-high-density lipoprotein cholesterol; PAD = peripheral artery disease; PCSK9 = proprotein convertase subtilisin/kexin type 9; TC = total cholesterol; TG = triglyceride. * *p* value was calculated when HLP5 vs. HLP4.

## Data Availability

Not applicable.

## Supplementary Materials

The following supporting information can be downloaded at: https://www.mdpi.com/article/10.3390/jpm13010068/s1. Table S1: Diagnostic criteria and possible pathogenic mechanisms of each FLL type of HLP in the present study. Table S2: Study variables in the present study. Figure S1: Illustration of plasma VLDL-C and percentages of change in plasma VLDL-C among patients.
