# Supplementary material for: Management of Patients with Type V Hyperlipoproteinemia: An Uncommon Phenotype of Dyslipidemia with Chylomicronemia and Severe Hypertriglyceridemia
Source: J Pers Med. 2022 Dec 28;13(1):68. doi: 10.3390/jpm13010068 (PMC9866642; doi:10.3390/jpm13010068)
Supplement: Supplementary file 1 [file jpm-13-00068-s001.zip › jpm-2082254-supplementary.pdf]

Supplementary Material

**Table S1. Diagnostic criteria and possible pathogenic mechanisms of each FLL type of HLP in the present study***		
**Type**	**Diagnostic criteria****	**Possible pathogenic mechanisms**
**Type 1 (HLP1)**	- ApoB <75 mg/dL<br>- TG ≥150 mg/dL<br>- TG/apoB ratio ≥8.8<br>- Presence of chylomicrons on LPEP	HLP1 is a very rare disorder with homozygous mutation in *LPL, GHIHBP1, APOC2, APOA5,* or *LMF1* genes and deficiency of lipoprotein lipase (LPL).
**Type 2a (HLP2a)**	- ApoB ≥120 mg/dL<br>- TG <150 mg/dL<br>- Absence of chylomicrons on LPEP	HLP2a is a common polygenic lipid disorder with hypercholesterolemia and high LDL-C which may be associated with dietary and environmental factors. However, the most severe presentation of HLP2a is familial hypercholesterolemia which is resulted from homozygous or heterozygous mutation in *LDLR, APOB,* or *PCSK9* genes.
**Type 2b (HLP2b)**	- ApoB ≥120 mg/dL<br>- TG ≥150 mg/dL<br>- Absence of chylomicrons on LPEP	HLP2b is a common polygenic lipid disorder with hypercholesterolemia and mild hypertriglyceridemia (HTG) which is usually associated with dietary and environmental factors.
**Type 3 (HLP3)**	- ApoB ≥120 mg/dL<br>- TG/apoB ratio <8.8<br>- TC/apoB ratio ≥2.4<br>- The presence of broad-β band on LPEP	The cause of HLP3 is a specific presentation (ε2/ε2) of *APOE* gene polymorphism.
**Type 4 (HLP4)**	- ApoB <120 mg/dL<br>- TG ≥150 mg/dL<br>- TG/apoB ratio <8.8<br>- TC/apoB ratio <2.4<br>- Absence of chylomicrons on LPEP	HLP4 is a very common presentation of lipid disorder and mainly caused by metabolic disorders (e.g., obesity, insulin resistance, and diabetes).
**Type 5 (HLP5)**	- ApoB <120 mg/dL and ≥75 mg/dL<br>- TG ≥150 mg/dL<br>- TG/apoB ratio ≥8.8<br>- Presence of chylomicrons on LPEP	HLP5 is caused by heterozygous mutation in LPL, GHIHBP1, APOC2, APOA5, or LMF1 genes, or burdens of single nucleotide polymorphisms in multiple TG-associated genes (i.e., *DOCK7, ANGPTL3, GALNT2, KLHL8, AFF1, MAP3K1, ANKRD55, MLXIPL, NAT2, TRIB1, JMJD1C, FADS1, FADS2, FADS3, APOA1, APOC3, APOA4, CAPN3,*

		FRMD5, CETP, SUGP1, PLTP, GCKR, CYP26A1, CILP2) accompanied by secondary metabolic or environmental factors (i.e., obesity, alcohol drinking, or diabetes).

Abbreviations: ApoB = apolipoprotein B; FLL = Fredrickson, Levy, and Lees; HLP = hyperlipoproteinemia; LPEP = lipoprotein electrophoresis; TC = total cholesterol; TG = triglyceride; VLDL = very-low density lipoprotein.

*The unit of each lipid parameter used in the present study was mg/dL.

**Each characteristic should be met.

Table S2. Study variables in the present study	
**Variables**	**Definition and description**
**Age**	
**Sex**	Male or female
**Weight**	Unit: kilogram
**Height**	Unit: cm
**Smoking habit**	Current smoker
**Body mass index**	Weight in kilograms divided by the square of height in meters
**Coronary artery disease (CAD)**	defined as those who had > 50% diameter stenosis of major epicardial coronary arteries confirmed by coronary computed tomography (CT) angiography or coronary angiography
**Ischemic stroke (IS)**	Determined by the neurologist's records with confirmation by brain CT or magnetic resonance imaging
**Peripheral arterial disease (PAD)**	Confirmed by ankle-brachial index < 0.9 or > 1.4 and/or > 50% diameter stenosis of peripheral arteries observed in CT angiography
**Diabetes mellitus (DM)**	Determined by medical records and prescribed medications
**Hypertension (HTN)**	
**Pancreatitis**	Identified by patients' admission records together with detection of raised circulating pancreatic enzyme and pancreatic CT scans
**Prescribed medications**	Antiplatelet, beta-blocker, angiotensin con-verting enzyme inhibitor/angiotensin receptor blocker (ACEI/ARB), and lipid-lowering therapy (LLT) including statin, ezetimibe, proprotein convertase subtilisin/kexin type 9 (PCSK9) inhibitor, fenofibrate, niacin, and omega-3 fatty acid

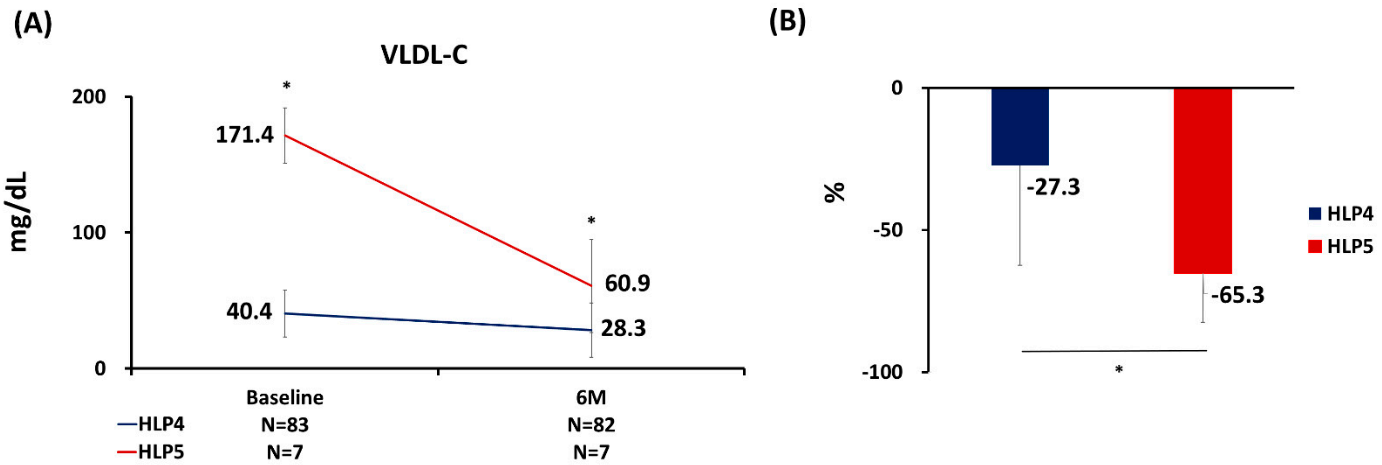

Figure S1. Illustration of plasma VLDL-C and percentages of change in plasma VLDL-C among patients. (A) Plasma VLDL-C. (B) Percentages of change in plasma VLDL-C. HLP4 = type IV hyperlipoproteinemia; HLP5 = type V hyperlipoproteinemia; N = numbers; VLDL-C = very-low-density lipoprotein cholesterol.
